# Statement of Retraction: The regulation of cell homeostasis and antiviral innate immunity by autophagy during classical swine fever virus infection

**DOI:** 10.1080/22221751.2025.2595753

**Published:** 2025-12-09

We, the Editors and Publisher of Emerging Microbes & Infections, have retracted the following article:

Li, X., Song, Y., Wang, X., Fu, C., Zhao, F., Zou, L., … Fan, S. (2023). The regulation of cell homeostasis and antiviral innate immunity by autophagy during classical swine fever virus infection. Emerging Microbes & Infections, 12(1). https://doi.org/10.1080/22221751.2022.2164217

After the publication, the authors identified errors in the Western Blot figures, and the following correction was published:

Correction. (2025). *Emerging Microbes & Infections*, *14*(1). https://doi.org/10.1080/22221751.2024.2445896

Following the correction, further concerns were identified by the publisher regarding image integrity of Western Blot Figures 4A, 4B, 5D, 6A, 6B,6D, 6E, 6F,6H, 7A, 7B, 8C, 8D, 8E, 8F, and RT-PCR methodology. When approached for an explanation, the authors provided original data, however, concerns remain.

As verifying the validity of published work is core to the integrity of the scholarly record, we are therefore retracting the article. The authors have been informed.

We have been informed in our decision-making by our editorial policies and the COPE guidelines.

The retracted article will remain online to maintain the scholarly record, but it will be digitally watermarked on each page as ‘Retracted’.

